# Determination of the Readability Level of Consent Forms Used in the Gynecology and Obstetrics Clinic at Suleyman Demirel University

**DOI:** 10.7759/cureus.37147

**Published:** 2023-04-05

**Authors:** Cem Dağdelen, Evrim Erdemoğlu

**Affiliations:** 1 Clinic of Obstetrics and Gynecology, Isparta Private Meddem Hospital, Isparta, TUR; 2 Department of Gynecologic Oncology, Suleyman Demirel University, Isparta, TUR

**Keywords:** valid informed consent, medical ethics, women and health, consent forms, readability

## Abstract

Background

This study aimed to evaluate the readability level of consent forms used for interventional procedures in the obstetrics and gynecology clinic and to determine the readability of the texts according to the education level of the patient.

Methodology

This study determined the readability of patient consent forms used before interventional procedures in the gynecology and obstetrics clinic at the Suleyman Demirel University Hospital, Isparta. The consent forms were divided into two main groups according to their use in obstetrics and gynecology procedures. The readability level of consent forms was assessed using two readability formulas developed by Ateşman and Bezirci-Yılmaz, which determine the readability level of Turkish texts in the literature.

Results

When the consent forms were analyzed according to Atesman’s readability formula, they were found to be readable with more than 15 years of education at the undergraduate level, while according to Bezirci-Yılmaz’s readability formula, they were found to be readable with 17 years of education at the postgraduate level.

Conclusions

Easy-to-read consent forms will ensure that patients are more informed about interventional procedures and participate more effectively in the treatment process. There is a need to develop readable consent forms suitable for the general education level.

## Introduction

Informed consent is a fundamental ethical and legal requirement that ensures an individual has full awareness of the procedure they will undergo and their agreement to it. There has been much discussion around informed consent from ethical and legal perspectives. It is widely recognized that verbal information beyond what is normative cannot constitute informed consent, while consent obtained without precise details is deemed invalid. In cases where the patient is unable to meet the competence requirements to make their own medical decisions (such as individuals under the age of 18, those with insufficient mental capacity, or those under guardianship), informed consent can be obtained from their relatives or a legal representative designated as their guardian [1].

The notion of readability is used in informed consent forms to assess the comprehensibility of written texts. It is possible to determine the readability and suitability of a text according to the degree of education by estimating the number of words and syllables in a written text using various mathematical methods.

The importance of informed consent is better understood given the threatening impact of malpractice lawsuits against physicians, where interventional procedures are often performed in obstetrics and gynecology clinics. However, clinical consent procedures often prove inadequate. Reasons for a flawed consent process include difficulty in reading consent forms and a patient’s insufficient level of education.

This study aimed to evaluate the readability of consent forms used for interventional procedures at the Suleyman Demirel University Hospital (SDUH), Isparta, Turkey, a tertiary center, and to determine how readable the texts are based on a patient’s level of education.

## Materials and methods

Parameters such as the word numbers in sentences and the syllable numbers in words play a role in determining the readability level. There are numerous formulas in which these parameters are used [2,3].

In this study, 18 consent forms were included, which are actively used in the clinic of gynecology and obstetrics at SDUH as of 2022. While eight of these consent forms were used during obstetric procedures, 10 were used during gynecological procedures. All consent forms used in our clinic were included in the study. All consent forms were in Turkish. The two main readability formulas that determine the level of readability of Turkish texts in the literature are the formulas developed by Ateshman and Bezirci-Yılmaz [4,5].

Ateshman readability score is calculated as 198.825 − 40.175 × (total syllables/total words) − 2.610 × (total words/total sentences). Bezirci-yılmaz readability score is calculated as = √AWN × ((S3 × 0.84) + (S4 × 1.5) + (S5 × 3.5) + (S6 × 26.25)) (AWN: average word number; S3: number of words with an average of three syllables; S4: number of words with an average of four syllables; S5: number of words with an average of five syllables; S6: number of words with an average of six or more syllables).

In Ateshman’s readability formula, the readability score ranges from 0 to 100. The higher the score, the easier it is to read the text (Table 1).

**Table 1:** The level of education according to the Ateşman readability formula.

Score	Education level
90–100	Fourth grade
80–89	Fifth or sixth grade
70–79	Seventh or eighth grade
60–69	Ninth or tenth grade
50–59	Eleventh or twelfth grade
40–49	Thirteenth and fifteenth grade
30–39	Bachelor’s degree
≤29	Academic level

In the readability formula by Bezirci-Yılmaz, the level of text readability according to the Turkish education system is determined using the above formula, which uses calculations based on the number of words and syllables in sentences (Table 2).

**Table 2:** The level of education to Bezirci-Yılmaz readability formula.

Grade	Education level
1–8	Primary education
9–12	Secondary education (high school)
12–16	Bachelor’s degree
16+	Academic level

Consent forms

Before all interventional procedures, patients are informed about the possible benefits of the procedure and complications arising from the interventional procedure, as well as the risks that may occur during the application of anesthesia during the procedure. Informed consent of the patient through the consent form should be obtained from the patients themselves, or, in cases where the patient is incompetent, from the patient’s relatives or legal representative. This study determined the readability of patient consent forms used before interventional procedures in the clinic of gynecology and obstetrics at the SDUH. The consent forms were divided into two main groups according to their use in obstetrics and gynecology procedures.

Statistical analysis

This is a cross-sectional, descriptive study. Using the Shapiro-Wilk test, the normal distribution of the data was assessed. Student’s t-test was used to compare the paired groups. Moreover, p-values <0.05 were accepted as the level of statistical significance.

Ethics declaration

Animal and human models were not used in this study; only consent forms were used. The study was approved by the SDU Local Ethics Committee (approval number: 168, dated 08.06.2022).

## Results

When all consent forms were analyzed, the average score according to the Ateshman readability formula was 38.6. Accordingly, the consent forms were readable with undergraduate-level education. The average Bezirci-Yılmaz readability score was 17.3, indicating that academic-level education was required. According to the Ateshman readability formula, four consent forms were found readable for the academic education level (16th grade and above), and 11 consent forms were found readable for the undergraduate education level (Table 3). Three consent forms were found readable for the secondary education level. None of the consent forms were found readable for the primary education level. According to the Bezirci-Yılmaz readability formula, 12 consent forms were found readable for the academic level, four were found readable for the undergraduate level, and two were found readable for those with 9-12 years of education (Table 3).

**Table 3:** Distribution of consent forms according to Ateşman and Bezirci-Yılmaz readability levels (N = 18).

	Primary education (4, 5, 6, 7, 8)	Secondary education (high school) (9, 10, 11, 12)	Bachelor’s degree (13, 14, 15)	Academic level (+16)
Ateşman formula	0	3	11	4
Bezirci–Yılmaz formula	0	2	4	12

According to both readability formulas, no consent forms were readable for the primary education level.

According to the readability formula by Bezirci, obstetrics consent forms could be read with an undergraduate education level with a value of 15.33 ± 3.74, while gynecology consent forms could be read with an academic education level with a value of 18.98 ± 2.14 (Table 4). Again, when obstetrics and gynecology consent forms were calculated using the Bezirci-Yılmaz and Ateshman readability formula, it was observed that longer education periods were required for the readability of gynecology consent forms (p = 0.019 and 0.001, respectively) (Table 4).

**Table 4:** Mean readability scores for consent form groups.

	Group	Mean readability scores	Mean ± SD	P-value
Bezirci–Yılmaz formula	Obstetrics consent forms (n = 8)	15.3	15.3 ± 3.7	0.019
	Gynecology consent forms (n = 10)	18.9	18.9 ± 2.1	
Ateşman formula	Obstetrics consent forms (n = 8)	44.9	44.9 ± 7.4	0.001
	Gynecology consent forms (n = 10)	33.5	33.5 ± 4.9	

## Discussion

Informed consent is a fundamental component of the ethical and clinical relationship [6]. When obtaining informed consent from a patient (the consent of a patient representative if the patient cannot make a decision or refuses to participate in the decision-making process), physicians should examine the ability of the patient to understand relevant medical information and the consequences of treatment alternatives, as well as the ability of the patient to make an independent, voluntary decision. Physicians should present relevant information accurately and sensitively following the patient’s preferences for receiving medical information and explain the purpose and nature of proposed interventions and treatment alternatives, including options for non-operative treatment during the consent for surgery. The risks, burdens, and expected benefits of all options, including stopping the treatment, informed consent discussion, and the patient’s (or representative’s) decision should be recorded in the medical record. When the patient or representative gives a specific written consent form, it should be included in the medical record [7]. Individuals demonstrate the capacity to make decisions when they can understand the benefits, risks, and alternatives of treatment options and their clinical condition. Therefore, it is crucial to communicate a choice clearly and coherently, reasoning logically over options and possible outcomes [8]. Patients show that they approve of these choices in their consent forms. At this stage, the importance of the readability of the texts in consent forms increases. The term readability refers to the level of education to which the written text is appropriate. Readability formulas, informed consent, behavioral therapy guidelines, and psychological tests are increasingly applied to measuring written materials in the field [9]. Although readability tests do not give definitive results about the comprehensibility of the text, they provide an idea about the level of the text [10].

The first readability formula was produced by Flesch in the first half of the twentieth century [11]. The number of studies using readability formulas in Turkish medical texts is extremely small, and the two main formulas used are those developed by Ateşman and Bezirci-Yılmaz.

A study by Ay et al. on the readability of 75 different drug package inserts found that eye drop package inserts could be read with an average undergraduate-level education [12]. In a study on the readability of websites, Solak et al. examined 1,250 websites for the readability of websites containing information about colorectal cancer. They found that the readability level of the information texts on the existing websites was at the undergraduate level [13].

As the average person in Turkey has seven to eight years of education, it is more appropriate to write consent forms at least according to the fifth and sixth-grade level [14]. According to the average education level in Turkey, the readability level of the consent forms evaluated in this study requires a very high level of education. According to both readability formulas, the readability of the gynecological consent forms was more difficult. Writing consent forms according to the fifth and sixth-grade levels will improve the informed consent process.

We could not find any other study in the literature examining the readability of consent forms used in obstetrics and gynecology clinics. In another study on readability evaluated using the formulas of Ateşman and Bezirci-Yılmaz, Ebem et al. examined 90 separate intramuscular and intravenous injection informed consent forms. The study found that these forms could be read with secondary education [15].

Furthermore, in the study by Ay et al. on patient consent forms for ophthalmologic interventional procedures, ophthalmologic consent forms were found readable with at least 11 years of education according to Ateshman and Bezirci-Yılmaz readability formulas [16].

In a study conducted by Eryılmaz et al. which aimed to determine the readability levels of skin cancer patient information texts, in the analysis of 74 websites, the average readability level was calculated as medium difficulty (high school education level) according to Ateshman and as undergraduate level according to Bezirci-Yılmaz. In the study by Eryılmaz et al., the information texts in question were more accessible to read than the consent forms included in this study [17].

In a study by the National Institutes of Health and American Medical Association on the level of readability of consent forms that are used before invasive procedures in which seven different readability formulas were used, it was found that the consent forms were recommended to be readable with an education at the sixth-grade level. In contrast, consent forms used in invasive procedures were found readable with an education at the 15th-grade level on average [18].

In recent years, the number of studies evaluating consent forms, health-related websites, and drug prospectuses has increased in studies on readability. We did not find a study where consent forms used in obstetrics and gynecology clinics were evaluated with Turkish legibility formulas.

According to the results obtained using the Ateshman formula, a bachelor’s level education is required (Table 4). On the other hand, when consent forms were evaluated with the Bezirci-Yilmaz readability formula, it was seen that the obstetrics and gynecology consent forms were readable with the academic level of education.

Factors such as patients’ emotional state, additional medical conditions, and the font and font size of consent forms can also affect readability during the consent process. These factors were not considered, and we can consider this a limitation of our study.

## Conclusions

This study showed that the readability of the consent forms evaluated was higher than the general education level. According to this research conducted in a tertiary hospital, consent forms in the field of obstetrics and gynecology were found to have very low readability levels. These texts are required to have a readability level that is easier to understand for the public. There is a need to develop consent forms with readability appropriate for the general education level.
